# Concomitant primary angiosarcoma and invasive carcinoma of the breast: a case report

**DOI:** 10.11604/pamj.2022.42.70.31484

**Published:** 2022-05-25

**Authors:** Mouna Zghal, Meriam Triki, Fatma Khanfir, Marwa Bouhamed, Imen Mallouli, Doulira Louati, Tahya Boudawara, Ines Saguem

**Affiliations:** 1 Pathology Department, Habib Bourguiba University Hospital, Sfax, Tunisia,; 2 Gynecology and Obstetrics Department, Hedi Chaker University Hospital, Sfax, Tunisia

**Keywords:** Breast, primary angiosarcoma, invasive carcinoma, collision tumor, case report

## Abstract

Collision tumor is a rare entity composed of two different tumors that occur in close to one another and maintain distinct borders. Only few cases have been reported in the breast. We report the first case of concomitant and adjacent primary angiosarcoma (PBAS) and invasive carcinoma of the breast (IBC), in a 45-year-old patient which presented with a lump in her right breast. Biopsy revealed PBAS. She underwent mastectomy. Gross examination showed a hemorrhagic and spongy tumor in contact with a second small grayish-white mass. Histologically, the hemorrhagic tumor was consistent with a high grade (HG) PBAS; the second mass was consistent with an IBC with no images of histological admixture. The diagnosis of a collision tumor composed of HG PBAS and IBC was established. During follow-up, the patient developed ovarian angiosarcomatous metastasis. The diagnosis of breast collision tumor is very uncommon and hence is challenging for pathologist. Careful gross and microscopic examinations help in establishing the appropriate diagnosis.

## Introduction

Collision tumors are rare clinical entities in which two histologically distinct tumor types show involvement at the same site. The occurrence of these tumors in the breast is extremely rare. Concomitant primary angiosarcoma (PBAS) and invasive carcinoma in the same breast is exceptional and have been reported in only three cases previously [1-3]. In all cases, the two tumors were distant. We report an exceptional case of concomitant and adjacent angiosarcoma and invasive carcinoma of non-special type (IBC NST) in the female breast with detailed clinico-pathological features, treatment and outcome. This exceptional case was challenging for the pathologists because, two principal diagnoses can be established with different treatment modalities and outcome.

## Patient and observation

**Patient information:** a 45-year-old female, with no medical history, presented with a painful lump in her right breast.

**Clinical findings:** on examination, a mass measuring 8 cm in diameter was found in the lower quadrant.

**Timeline of current episode:** June 2020: ultrasound and biopsy examination. August 2020: a mastectomy was performed. October 2020: a complementary axillary dissection was made. January 2021: thoracic and abdominal computed tomography was performed. March 2021: ovariohysterectomy.

**Figure 1:**
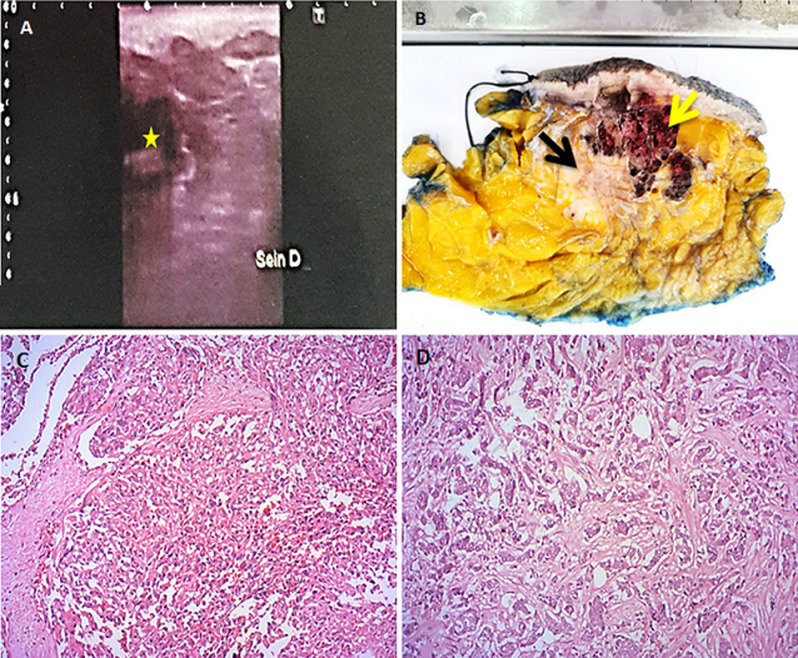
A) ultrasound image of the right breast: nodule with heterogenous echostructure (yellow asterisk); B) gross examination of the two adjacent tumors: spongy and hemorrhagic well-defined tumor (yellow arrow) adjacent to a second grayish-white and irregular tumor (black arrow); C) angiosarcomatous proliferation: anastomosing and irregular vessels with intraluminal tufting (HEx200); D) area of invasive carcinoma of no special type: cords and glandular structures composed of atypical cells (HEx200)

**Figure 2:**
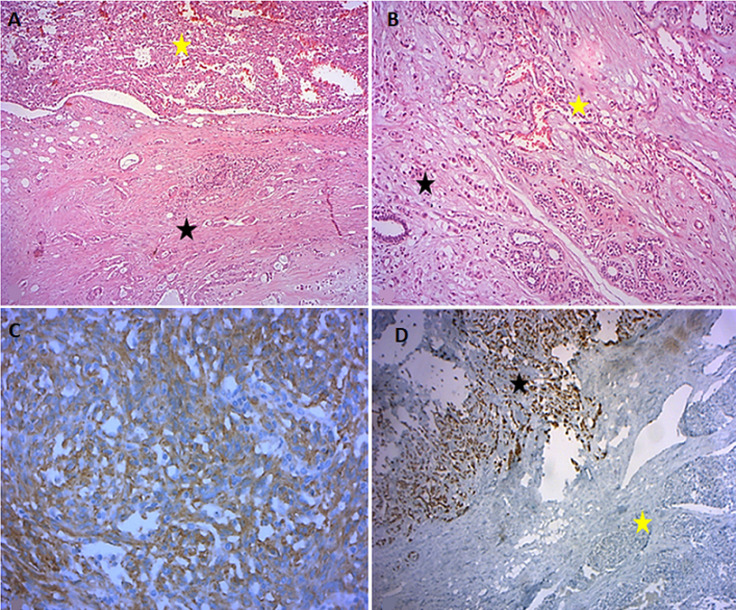
histological feature of the two adjacent tumors: angiosarcomatous proliferation (yellow asterisk) in contact with an invasive carcinoma (black asterisk) without histological admixture: A) low magnification (HEx100); B) high magnification (HEx200); C) CD31 immunohistochemical study: vascular proliferation positive for CD31 (x400); D) progesterone receptor immunohistochemical study: carcinomatous proliferation positive for progesterone receptor (black asterisk); vascular proliferation negative for progesterone receptor (yellow asterisk) (x100)

**Diagnostic assessment:** ultrasound examination disclosed a nodule with heterogeneous echostructure measuring 9.2 cm (Figure 1A). Biopsy revealed a mesenchymal proliferation with anatomizing vascular channels lined by atypical endothelial cells admixed with areas of atypical spindle cells positive for CD31. The findings were consistent with PBAS. The patient underwent a right radical mastectomy. At gross examination, there was a spongy and hemorrhagic tumor measuring 8.5 cm, in contact with a second grayish-white tumor measuring 3 cm (Figure 1B). Microscopically, the spongy tumor showed findings consistent with high grade (HG) PBAS (Figure 1C). This tumor was directly in contact with a second tumor composed of an epithelial proliferation arranged in clusters, cords and glands corresponding macroscopically to the grayish-white mass. Tumor cells were cuboidal with hyperchromatic and voluminous nuclei (Figure 1D). No images of intermingled epithelial and vascular proliferations were seen (Figure 2 (A,B)). Immunohistochemical analysis showed CD31 and CD34 positivity of the vascular proliferation. The epithelial proliferation was positive for hormonal receptors and negative for Her2, p63 and CK5/6. Ki-67 was 10% (Figure 2 (C,D)).

**Diagnosis:** after consultation with pathologists and careful search of the literature, the diagnosis of a collision tumor composed of HG PBAS and IBC NST was established.

**Therapeutic interventions:** a complementary axillary dissection was made and revealed lymph node metastasis of the IBC NST. The patient is scheduled for chemotherapy.

**Follow-up and outcome of interventions:** during follow-up, the patient developed hepatic and ovarian vascular lesions. Histological analysis confirmed the presence of ovarian angiosarcomatous metastasis.

**Informed consent:** the authors certify that they have obtained all appropriate patient consent forms. In the form, the patient has given his consent for her clinical information to be reported in the journal.

## Discussion

We presented a case of adjacent carcinomatous and sarcomatous proliferations in the female breast. Because of its small size, the carcinoma was incidentally discovered on the mastectomy specimen. Two main diagnoses were suggested. The first one was collision tumor. The second one was metaplastic carcinoma with angiosarcomatous metaplasia (MC). However, the presence of two independent tumors macroscopically, the absence of intermingled areas of the two proliferations histologically, and the immunophenotype of the invasive carcinoma which was not consistent with the usual triple-negative immunophenotype of MC with positivity of p63 and CK5/6, ruled out the diagnosis of MC. Moreover, the presence of metastases of a unique tumor type without intertwined carcinomatous and angiosarcomatous proliferations supported the diagnosis of collision tumour.

Collision tumor is a rare entity composed of two different tumors that occur in close to one another and maintain distinct borders [4]. Collision tumor of the breast are extremely rare and only few cases have been reported in the literature [5]. The particularity in our case was the presentation of PBAS and IBC NST as a collision tumor. In our knowledge, no similar cases have been described previously.

Collision tumor´s pathogenesis remains unclear. The first theory suggests that the neoplastic heterogenicity results from two different cell lines proliferation. This can be explained by the interaction theory: the damages caused by one neoplasm can induce the development of a second tumor. Other proposed the theory of hybrid neoplastic cell that derived from two stem cell precursor and would differentiate into two components. Other possible mechanism is the pure coincidence [4].

The specific treatment of collision tumor is difficult to assess. Some authors suggested combination of therapies, treating each tumor as if it were unique [6]. Others recommended focus management on the more aggressive tumor [4].

## Conclusion

Although rare, pathologists should consider the possibility of a breast collision tumor when encountering two adjacent epithelial and mesenchymatous proliferations.
